# Molecular Markers in the Pathogenesis of Cholangiocarcinoma: Potential for Early Detection and Selection of Appropriate Treatment

**DOI:** 10.4021/gr2009.06.1299

**Published:** 2009-05-20

**Authors:** Cornelia Braicu, Claudia Burz, Ioana Berindan-Neagoe, Ovidiu Balacescu, Marcel Tantau, Victor Cristea, Alexandru Irimie

**Affiliations:** a Cancer Institut “I Chiricuta”, Cluj-Napoca, Romania; b University of Medicine and Pharmacy “Iuliu Hatieganu”, Cluj-Napoca, Romania

**Keywords:** Cancer, Cholangiocarcinoma, Markers, Target therapies

## Abstract

Cholangiocarcinoma (CC) is a primary malignancy that arises from cholangiocytes, the epithelial cells lining the bile duct livers. The worldwide incidence of CC is increasing and despite of combined therapeutic strategies, its prognosis remains poor. Till now surgery remains the only curative treatment modality. Over the past years, several important studies have yielded new insights into the molecular mechanisms of cholangiocarcinoma. This review focused on critical molecular player during the development from inflammation and cellular and molecular pathogenesis of this disease. The novel prophylactic and therapeutic approach deals especially the molecules involved in inflammation of cholangiocite or those related to promotion and progression of CC. The elucidation of their specific effects and interaction of this complex mechanism will accelerate the development of new biomarker for early detection and predictor factors outcome in CC.

## Introduction

Cholangiocarcinoma (CC) is a fatal neoplasm that arises from cholangiocytes, the epithelial cells lining the bile duct liver. CC is the second most common primary liver cancer after hepatocellular cancer [1, 2]. CC occurs in approximately 2 per 100,000 people and accounts for approximately 13% of primary liver cancers [2, 3]. The prevalence of CC shows a wide geographical variety with the highest rates in Asia [1, 4] and the lowest rate in Australia [3]. Epidemiologic studies suggest an increasing incidence in the Western countries and Unites States [3, 4, 5]. According to American Society Cancer about 2,000 to 3,000 people in the United States develop bile duct cancer each year [4, 6].

The etiologies of CC remains unknown, but in most cases CC are associated with chronic biliary inflammation, cellular injury of bile ducts together with obstruction of bile flow [6, 7]. High risk-conditions in CC development are manifested thought: congenital biliary anomalies, primary sclerosing cholangitis, hepatolithiasis and hepatobiliary flukes (e.g. *Clonorchis sinensis,* and *Opisthorchis viverrini*). Other risk factors for cholangiocarcinoma include environmental toxins such as dioxin, and vinyl chloride, nitrosamines [4].

CC is an epithelial cancer of the biliary duct system that may originate in the liver and extrahepatic bile ducts, which may terminate at the ampulla of Vater. Based upon anatomic location, CC can be divided into three categories: (1) intrahepatic CC (20-25%), occurring in the bile ducts residing within the liver; (2) extrahepatic or perihilar CC (also known as Klatskin tumour, 50%), occurring at the confluence of the right and left hepatic ducts; and (3) distal extrahepatic bile duct cancers (20-25%) [2]. More than 90% of CCs are adenocarcinomas, with different histological variants including adenocarcinoma, papillary adenocarcinoma, intestinal-type adenocarcinoma, and mucinous adenocarcinoma [1, 2, 6].

Conventional chemotherapy and radiation therapy have not been shown to be effective in prolonging long-term survival and although photodynamic therapy has been reported to be effective as a palliative treatment, it is not curative [6]. Radical surgery is the only potentially curative treatment modality, but in most cases, the tumors are well advanced at the time of diagnosis, which results in limited treatment options [2], the impact of chemotherapy on survival remains controversial [4, 6]. The overall survival is approximately 6 months [4].

The poor response of cholangiocarcinoma to therapy highlights the need for increased efforts in understanding the etiology and pathogenesis of this primary liver cancer [4, 8]. More new strategies should be developed that allow detecting these tumors at early stage and permit to apply radical curative modalities [2, 4].

## Pathogenesis

**Figure 1:**
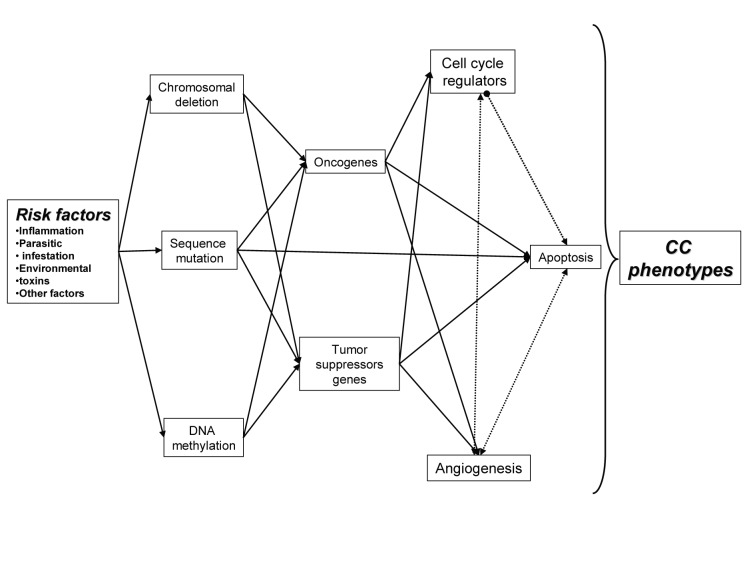
Relation between multiple factors behind CC, with important role in initiation and development of CC.

The development of cholangiocarcinoma, as with most cancers, is multifactorial process (Fig. 1). Contributers are thought to include chronic inflammatory processes that induce alteration of cellular detoxification mechanisms, induce activation of oncogenes, functional loss of tumor-suppressor genes and dysregulation of cell cycle, cell apoptotic mechanisms and angiogenesis [2, 4].

The persistent tissue damaging as well an enrichment or reactive oxygen and nitrogen species contribute to a cancer-prone microenvironment [7]. During inflammation, cholangiocites produce reactive oxygen species and other toxic compounds such as: nitric oxide, nitrate, nitrite, and oxygen radicals, that lead to death and regeneration of the bile ducts [7, 9]. This species are known to be DNA mutagens and are linked to malignant transformation. During this procees inflammatory processes, cholangiocites secrets mitogens that activate local cellular receptors and intracellular signalling pathways [4, 7, 9].

### Inflammation

The biliary tract inflammation underlines the pathogenesis in the most of the patients [3, 7, 9]. Chronic inflammation is thought to promote carcinogenesis by causing DNA damage [9], activating tissue reparative proliferation and by creating a local environment that is enriched with cytokines and other growth factors [2, 7].

The inflammatory cytokine interleukin-6 (IL-6) enhances tumor growth in CC by altered gene expression via autocrine mechanisms [10-12]. Interleukin-6 (IL-6)-mediated signal transducers activation is aberrantly sustained in cholangiocarcinoma cells, resulting in resistance to apoptosis [10, 11]. IL-6 expression is inversely related to cell proliferation and positively related to differentiation in CC [3, 12].

Suppressors of cytokine signalling-3 (SOCS-3) are a newly identified family of intracellular protein controlling the magnitude and/or duration of signals propagated by diverse cytokine receptors by suppressing their signal transduction process [11, 14, 15]. SOCS-3 epigenetic silencing is responsible for sustained IL-6/STAT-3 signalling in cholangiocarcinoma [10]. Recent reports suggest SOCS-3 may be silenced by epigenetic phenomenon in human cancers, namely methylation of CpG islands [10, 14]. Methylation of cytosine residues within promoter CpG islands is a well-established epigenetic process causing gene silencing. CpG island methylation is an attractive mechanism explaining sustained IL-6 signalling in human cholangiocarcinoma [10, 11, 13].

Tumor necrosis factor-a (TNF-a) is a mediator of inflammation with actions directed towards both tissue destruction and recovery. Accumulated evidence suggests that TNF-a may act as an endogenous tumor promoter in addition to its role in immune responses [16, 17]. It was observed overexpression of tumor necrosis factor receptors (TNFR) genes in CC associated with hepatolithiasis [3, 6, 16, 17].

Recently, there has been evidence for implication of chemokines migration in tumor dissemination. Among chemokines, CXC chemokine, stromal cell-derived factor-1 (SDF-1) (CXCL12), and its specific receptor CXCR4 have gained considerable interest because of their roles in carcinogenesis, being involved in the migration or invasion CC [17, 18].

Expression of cyclooxygenase-2 (Cox-2), an inducible enzyme controlling the synthesis of lipid inflammatory mediator prostaglandins, has been reported to be up-regulated in the malignant neoplastic epithelium of intrahepatic cholangionomas of both humans and experimental rodent models [17]. Additional induction of Cox-2 is mediated by bile acids, oxysterols, and iNOS [3]. Cox-2 plays an important role in the cholangiocarcinogenesis as a mediator of mitogenesis, anti-apoptosis and angiogenesis. However, the biologic function and molecular mechanisms of COX-2 in the control of cholangiocarcinoma cell growth have not been well established [18].

### Genetic and molecular abnormalities associated in CC

As in most of the cancers, multiple genes are involved in molecular transformation of normal functioning liver tissue to malignant cholangiocites [19]. Reactive oxygen species produced during inflammatory processes modify DNA bases and result DNA damage. This reactive species produced also alteration of key repair proteins of the DNA promoting the accumulation of potential oncogenic mutations important in the initiation and/or progression of CC [9].

#### Genetic and epigenetic changes

Genetic defects such as mutation and deletion can lead to the dysfunction of genes. Although epigenetic alteration does not change DNA sequence, it can affect the expression of genes by chemical modification including DNA methylation and histone deacetyleases [20, 21]. Recent data suggest that both genetic and epigenetic changes are required for transformation, promotion and progression of CC.

DNA methylation cooperates with histone deacetyleases in inhibiting transcription [21]. Methylation of multiple tumor suppressor genes is seen in cholangiocarcinoma [22]. The methylation profile of multiple genes in cholangiocarcinoma may facilitate the distinction of cholangiocarcinoma from benign biliary epithelium in clinical settings [23]. Hypermethylation of regulatory regions called CpG islands in some tumor suppressor genes induce their inactivation [21].

Therefore, understanding the molecular events associated with the neoplastic transformation of cholangiocytes to CC may aid in the development of improved therapeutic strategies [21, 22].

#### Cell cycle regulators

**Figure 2:**
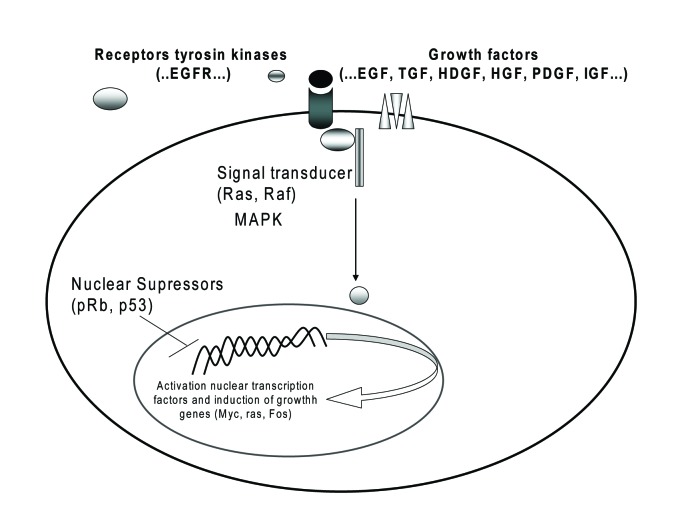
Oncogenes and their receptors involved in CC.

Cyclin and cyclin-dependent kinase complexes are involved in the cell cycle progression. Disruption of the G1/S and G2/M check points leads to uncontrolled cell growth, resulting in the development and progression of cancers (Fig. 2). Over-expressions of cyclins have been found correlate with the tumor relapse of human CC [2, 24]. Overexpression of cyclin B1 which acts at the G2/M phase checkpoint of the cell cycle has found in CC being associated with poor prognosis [20, 24]. Cyclin D1 is considered as oncogene and can promote progression of the cell cycle to S by cyclin D-dependent kinases (CDK4/CDK6)-mediated phosphorylation of the retinoblastoma (Rb) protein. Cyclin D1 overexpression was more frequently observed in cases of intrahepatic cholangiocarcinoma with poor or moderate differentiation and with lymph node metastasis [6]. Cyclin D1 seems to be involved in the carcinogenesis of both biliary intraepithelial neoplasia and intraductal papillary neoplasm of the bile duct [24]**.**

The *p53* tumor suppressor gene is the most common mutated gene in human cancer, occurring in approximately 50% cancers. In CC, p53 mutation has been shown to be present in 28-61% of patients [6, 25, 27]. Located on chromosome 17p13.1, p53 is responsible for cell cycle regulation at the G1/S and G2/M checkpoints. Inactivation of p53 caused by missens mutations or interaction with oncogenic viral proteins allows progression through the cell cycle without a physiological checkpoint and resulting from a selective growth advantage for cancer cells. Alteration of p53 gene plays a key role in late-stage events of tumor pathogenesis and is associated with poor prognosis of CC [25-27], but the others show no association of protein over-expression with outcomes, however the role of p53 remains controversial.

Alterations in p53 and p16I^NK4a^ are frequently detected in CC and are likely contributing to oncogenesis in the biliary tract. Point mutations in the promoter region of p16I^NK4a^ seem to represent an apparent early event associated CC [6].

The retinoblastoma gene encodes a protein, *Rb*, which blocks transition from G1 to S phase in cell cycle being necessary for prevention of cell replication when DNA has been damaged. The correlation between *Rb* expression and survival of patients with CC has not been proven [10, 24].

*DCP4* is a tumor suppressor gene involved in transforming growth factor b signalling pathway (TGF-b) [3, 30], TGF-b being a major cell proliferation inhibitor. Loss of DCP4 led to progression in the cell cycle from G1 to S phase with increasing proliferation. Mutational inactivation of DCP4/Smad4, has been found to occur more commonly in distal bile duct cancers (55-60%) than in proximal bile duct and intrahepatic tumors but there was no correlation with survival [20, 28].

It was studied the expression of transformation suppressor gene *RECK* (reversion-inducing-cysteine-rich protein with kazal motifs) in hilar cholangiocarcinomas and its clinical significance by using reverse transcription-polymerase reaction in 42 paraffin-embedded samples of hilar cholangiocarcinoma and 10 samples of benign bile duct diseases. The abnormal expression of RECK gene might be one of the molecular mechanisms of hilar cholangiocarcinoma metastasis [30]. Others tumor suppressor gene whose expression is modified in CC are p16, p27, p57, SMAD4, p16INK4a, p21*WAF1* [4, 6, 20].

#### Oncogenes and their receptors

Proto-oncogenes encode a wide range of proteins products involved in the control of cell proliferation and differentiation (Fig. 2), including growth factors, growth factors receptors, components of signal transduction pathways and transcription factors [4, 20].

In CC, like in many other malignancies, receptors of tyrosine kinase (RTK) and its ligands are overexpressed. The binding of RTK to their growth factors can induce homodimerize or heterodimerize of the receptors proteins and activate different molecules that encode and regulate cell diferentioation, cell proliferation, cell survial and angiogenesis [3, 19, 20, 29]. RTK appear to be considered as important protooncogenes for cholangiocarcinogenesis, being used as pharmaceutically targeted.

The trans-membrane RTK of the epidermal growth facteor receptor (EGFR) family plays a significant role in cellular growth and proliferation signalling. Activation of EGFR and its ligands, transforming growth factor alpha (TGF-a) are hypothesized to form an autocrine growth loop and initiates a series of signal transduction cascades that include mitogen-activated protein kinase (MAPK), Akt, and other enzymes [31]. Inhibition of EGFR signaling has been shown to significantly suppress cholangiocarcinoma cell growth [3, 25, 29]. Generally, TGF-a is overexpressed in CC cells [2], more commonly in distal bile duct cancers than in more proximal bile duct and intrahepatic tumors. TGF-β1 expression is low in normal intrahepatic biliary cells, but markedly is increase in inflammatory and obstructive lesions of the bile duct. There are also reports suggesting that the TGF-β1 signaling system plays a role in carcinogenesis and cancer progression [3, 28].

The c-erbB-2 proto-oncogene encodes a transmembrane protein which is homologous to the EGFR. Overexpression of c-erbB-2 protein has been reported in many human carcinomas including CC and also in noncancerous biliary proliferative lesions such as hepatolithiasis. These suggest that c-erbB-2 oncogene participates not only in cholangiocarcinogenesis but also in biliary cell proliferation in non-neoplastic conditions [29, 30].

While it now seems apparent that aberrant EGFR and/or ErbB2 expression and signaling is associated with the molecular pathogenesis of intrahepatic cholangiocarcinoma, there is still a significant gap in our knowledge as how to best exploit such alterations in terms of targeted therapies that can then be successfully translated into positive clinical outcomes.

c-Met is a heterodimeric tyrosine kinase receptor for HGF. It is overexpressed especially in well-differentiated CC being liked to cell invasion, angiogenesis, and tumor differentiation/proliferation [19, 33]. Hepatocyte growth factor (HGF) regulates diverse biological responses including cells proliferation. HGF is a mitogenic factor and its overexpression has found in CC [3]. HGF activates both proliferation and invasion machinery in CC cells, suggesting that HGF might promote their malignant behaviour by concomitant activation of different biological functions [33]**.** c-Met and HGF antibody directed therapies receptor interaction have been shown biological activity in animal models and human studies [19]. A large number of c-Met tyrosine kinases inhibitors have examined, some of this inhibitors completing Phase I trials and beginning Phase II trials in humans. Due to their side effect have a limited application in CC treatment [19].

Recently was shown that CC express estrogens receptor (ER–α and –β) directly linked with insulin-like growth factor 1 (IGF1) and IGF1-R (receptor) [21], which could represent a strategy for the management of cholangiocarcinoma. Expression of other growth factors like: platelet–derived growth factor (PDGF), and hepatoma-derived growth factor (HDGF) have shown to be altered during cholangiocarcinogenesis [2, 28].

The *k-ras* oncogene encodes a family of signal transduction proteins downstream of growth factor receptors. Mutations of k-ras gene activate cellular proliferation and promote cellular growth. K-ras mutations, typically at codon 12, have been reported to be less frequently detected in peripheral CC than in hilar CC. The incidence of K-ras mutations has been reported to be higher in CC patients with lymph node metastasis than in those without lymph node metastasis but no significant correlation with survival has found [4, 6, 20].

#### Mucins

The transmembrane mucins (MUC) interact by different mechanisms with RTK family receptors and can activate signal transduction. MUC are high molecular weight glycoproteins synthesised by epithelial cells in many organs which form a protective barrier at the mucosal surface or act as transmembrane proteins [25, 29]. Meanwhile the cytoplasmic domain of mucins harbors several tyrosine residues that when phosphorylated may provide critical docking sites for initiating downstream cytoplasmic signaling pathways relevant to cancer development and progression. MUC1 and MUC2 have been shown to function as intramembrane ligand and modulator for ErbB-2. More recently, MUC1 have also shown to modulate TGF-a dependent CC progression but also was reported to facilitate neoplastic development by blocking activation of the intrinsic apoptotic pathways [25, 29].

MUC1 apomucin is frequently expressed in various subtypes of intrahepatic cholangiocarcinoma, including mass-forming and periductal infiltrating forms [6]. In contrast, MUC2, an intestinal-type mucin that is selectively expressed predominantly in well-differentiated and noninvasive mucinous-type intraductal cholangiocarcinomas with gastrointestinal differentiation, predicts a more favourable prognosis [29]. MUC2 is rarely expressed in invasive CC, but is expressed in cystadenocarcinoma [25]. MUC4 was also recently demonstrated to be an independent risk factor for poor prognosis in patients with the mass-forming type of intrahepatic cholangiocarcinoma. MUC5AC, which is a gastric-type mucin was not expressed in mass-forming cholangiocarcinoma [6, 25]. MUC6 showed a good correlation with the survival of CC patients, it may be used as prognostic marker for CC [34].

### Apoptosis - a tumour promotion factor

Failure of apoptosis is one of the key hallmarks of tumorogenesis. Apoptosis is regulated by two major pathways. One is the extrinsic pathway via death receptors on the cell surface and the other, the intrinsic pathway, depends on mitochondria and is initiated by cytochrome C release [6, 35, 36, 37].

The extrinsic pathway is initiated through the stimulation of the transmembrane death receptors, belong to the tumor necrosis factor superfamily formed by six death receptors including TNF receptor 1 (TNF-R1), Fas, DR3, DR6, TNF related apoptosis-inducing ligand receptor (TRAIL-R1, TRAIL-R2)**.** In recent years, the role of the Fas/Fas ligand (Fas/FasL) apoptotic signalling pathway in CC has been increasingly investigated [8, 35, 38]. Fas over-expression has observed in CC being associated with tumor differentiation. There are differences in the frequency of Fas expression between intrahepatic and extrahepatic bile duct cancers, which may affect tumor development by activation or deactivation of different tumor-promoting activities [6, 17, 39]. TRAIL expression was upregulated in preneoplastic disease, primary sclerosing cholangitis, human cholangiocarcinoma specimens and acts by promoting cell migration and invasion via a NF-kB-dependent pathway. Tumor necrosis factor related apoptosis-inducing ligand (TRAIL) is a promising agent for cancer therapy [20, 40].

The intrinsic pathway involved the mitochondria and is regulated by several families including Bcl-2 protein family. There are at least 20 proteins in the Bcl-2 family divided into pro-apoptotic (Bax, Bak, Bok, Bid, Bim, Noxa, Puma, Hrk) and antiapoptotic (Bcl-2, Mcl-1, Bcl-1, A1, Bcl-xL) members [8, 36, 37]. High concentrations of Bcl-2 or Bcl-xL affect the susceptibility of a cell to the induction of apoptosis by altering the ratio of death promoters to suppressors, providing tumour cells with a survival advantage, and permitting expansion of transformed cells harbouring mutations within their genome [36, 37]. Benign and neoplastic biliary epithelium co-expresses the cell survival proteins Bcl-XL and Mcl-1, but not Bcl-2. Through inhibiting apoptosis, the Bcl-xL and Mcl-1 proteins expressed by CC may be contributing to the low efficacy of chemotherapy and radiotherapy in this disease [4, 36, 40]. The role of the Bcl-2 family in CC remains to be established.

### Angiogenesis, invasion and metastasis

**Figure 3:**
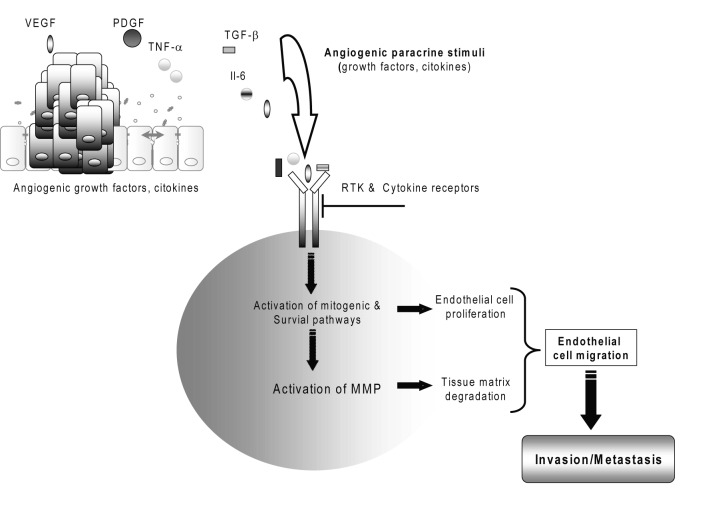
Cholangiocarcinoma tumor angiogenesis.

During tumor invasion, neovascularization (de novo formation of functional microvascular networks) and angiogenesis (pre-existing capillary extension) deliver nutrients and oxygen to malignant cells and help prevent the tumor mass from outgrowing the native vascular network (Fig. 3) [7, 41]. However, there are only few studies that have investigated this area in relation to the prognostic implications in CC [6, 42].

Vascular endothelial growth factor (VEGF or VEGF-A), as well as of the lymphangiogenic factor VEGF-C, has been detected to be overexpressed in the cancerous epithelium of a significant percentage of human intrahepatic cholangiocarcinomas and in human cholangiocarcinoma cell lines [41, 42]. VEGF may contribute to the “angiogenic switch” and malignant phenotype in human cholangiocarcinoma [29, 41]. Several angiogenesis-related factors including angiopoietin-1, angiopoietin-2 and thrombospondin-1 have been studied in CC but no correlation was found with survival [2, 4].

Both invasion and neovascularization require extracellular matrix breakdown and the subsequent migration of cells through the degraded structures [25]. Because extracellular matrix remodeling is the major activity of a family of enzymes known as matrix metalloproteinases (MMPs), these enzymes have come under investigation for their contributions to the malignant phenotype [43, 44]. Matrix metalloproteinase MMP-2, MMP-7 and MMP-9, regulated by tissue inhibitor of metalloproteinases TIMP-2, TIMP-7 and TIMP-9, respectively, play important roles in the degradation of the basement membrane during tumor invasion [25, 30, 43, 44]. The activities of MMP-2 and loss of balanced expressions of MMP-2/TIMP-2 and MMP-9/TIMP-1 are suggested as playing important roles in invasive growth related to the gross type of cholangiocarcinoma [45]. A matrix metalloproteinase inhibitor to treat unresectable cholangiocarcinoma has reported [43].

## Serum biomarkers for the enhanced detection of cholangiocarcinoma

CC is a fatal neoplasm and usually hard to get diagnosed in the early stage due to the unfavourable anatomic location. Therefore, early diagnosis, based on serum markers and the development of novel systemic therapies for advanced disease are very important.

Carcinoembryonic antigen (CEA) is a glycoprotein tumor marker meanwhile carbohydrate antigen 19-9 (CA19-9), is a mucin-type glycoprotein in serum being used for the diagnosis of malignancies in the stomach, colon and pancreas but also for bile duct cancers. Serum CA19-9 was proven to be superior to serum CEA in the diagnosis of cholangiocarcinoma and often considered the standard marker for pancreatic cancer and cholangiocarcinoma [46]. However, these markers are not always helpful, with sensitivity and specificity of approximately 70% and 50%, respectively [3].

Apolipoprotein A-II (ApoA-II) is the second most abundant protein in high-density lipoproteins (HDL) and is primarily synthesized by liver. In most studies, a constant steady-state level of ApoA-II is essential to maintain functional homeostatic regulation [20, 47]. There are an increasing number of reports suggesting a relationship between cancer susceptibility and the lipid metabolic pathway proteins, including apolipoproteins. Serum levels of ApoA-II were significantly elevated in CC but not other cancer types like oesophagus and ovarian cancers [47]. Some other serum markers, such as: bilirubin**,** C-reactive protein**,** sialic acid have been indicated to be available as supplementary markers to CEA and CA 19-9 [20, 47].

MUC1 and MUC4 have been reported as being independent diagnosis and prognostic markers for predicting poor outcomes in patients with mass-forming intrahepatic cholangiocarcinoma [29]. Serum MUC5AC may be used to enhance the diagnostic accuracy of CC [48].

Serum IL-6 concentration is proposed as novel biomarker for diagnosis of cholangiocarcinoma but also for monitoring the response to different therapies [12].

## Conclusions

Cholangiocarcinoma continues to be a challenging cancer that requires innovative approaches to permit early diagnosis or prevention in high-risk population. This type of cancer is increasing in its incidence worldwide and at present, there is no standard treatment for this fatal cancer. A better clarification of mechanism linking inflammation and cholangiocarcinogenesis will be beneficial in identification of molecules associated with cholangiocite inflammation, taking in account that is the main risk factor associated with this type of cancer.

The molecular mechanisms associated with cancer initiation and progression remains unclear. Further molecular characterization of CC may lead to earlier diagnosis, and the development of more effective therapies. In recent years, proteomics has become a widely developing technique in the field of biotechnology. A primary goal of proteomics is biomarker discovery for various human diseases, especially cancers, and plasma and serum are considered as the sources of choice in molecular diagnostics. The prognostic factors or diagnostic biomarkers of plasma or serum are important trends that deserve attention. Developing new blood biomarkers will help develop more effective therapeutic strategy targeting key signalling. In present are identified new target molecules involved in cholangiocarcinogensis that are in the research stage and are needed to be validate. One novel candidate as biomarker for early diagnosis is circulating IL-6.
